# Sevoflurane

**DOI:** 10.12688/f1000research.6288.1

**Published:** 2015-08-25

**Authors:** Stefan De Hert, Anneliese Moerman

**Affiliations:** 1 Department of Anesthesiology, Ghent University Hospital, De Pintelaan 185, Ghent, B-9000, Belgium

**Keywords:** Sevoflurane, anesthetic, anesthesiology, pharmacodynamic, pharmacokinetic

## Abstract

Sevoflurane has been available for clinical practice for about 20 years. Nowadays, its pharmacodynamic and pharmacokinetic properties together with its absence of major adverse side effects on the different organ systems have made this drug accepted worldwide as a safe and reliable anesthetic agent for clinical practice in various settings.

## Introduction

Although sevoflurane was synthesized in the early 1970s ^1^, it was not released for clinical use until the early 1990s. This was related partly to the expensive synthesis and the initial concern of apparent toxic effects ^2^, which later appeared to be a consequence of a flawed experimental design ^3^. Nowadays, its pharmacodynamic and pharmacokinetic properties together with its absence of major adverse side effects on the different organ systems have made this drug accepted worldwide as a safe and reliable anesthetic agent for clinical practice in various settings.

## Physicochemical properties

Sevoflurane (1,1,1,3,3,3-hexafluoro-2-(fluoromethoxy)propane) is a colorless, volatile, and non-flammable liquid with a characteristic smell. It is stable at room temperature and has a boiling point of 58.6°C and a vapor pressure of 157 mm Hg. Hence, in contrast to desflurane, it can be used in standard vaporizers ^3^. Sevoflurane has an oil/gas partition coefficient of 47.2 and its minimal alveolar concentration (MAC), which is the percentage that is necessary to prevent movement in 50% of patients during skin incision, is 2.05% ^4, 5^. As a consequence, its potency is considerably lower than that of the older inhalational agents such as halothane and isoflurane, but it is about three times more potent than desflurane.

Upon contact with alkaline carbon dioxide (CO _2_) absorbers, sevoflurane undergoes degradation ^6– 8^. The most important degradation product is fluoromethyl-2,2-difluoro-1-(trifluoromethyl) vinyl ether, better known as compound A. In experimental studies, compound A has been reported to be nephrotoxic ^9, 10^. Although the clinical implications of these findings remained unclear ^11^, the safety issue related to compound A formation led to intense debates for many years before the issue was resolved ^12^.

In 1996, Abbott Laboratories voluntarily recalled one lot of sevoflurane because evaluation of a bottle of sevoflurane revealed an uncharacteristically pungent odor ^13, 14^. This was caused by the formation of hydrogen fluoride as a consequence of a Lewis acid-fluorocarbon reaction. Even in minute amounts, this substance is highly reactive and toxic and can cause respiratory irritation and pulmonary hemorrhage ^15^. Subsequently, the offending Lewis acid (ferric oxide)-containing part was removed from the sevoflurane handling equipment, and a Lewis acid inhibitor (water) was added to the final product ^16, 17^. Although Abbott adapted the production process to create a “high water” sevoflurane (>300 parts per million), the manufacturers that subsequently launched sevoflurane (Minrad and Baxter) did not ^18– 20^.

## Pharmacological properties

It is beyond the scope of this review to discuss in detail the pharmacological properties of sevoflurane. Several excellent review articles have addressed this topic ^21– 25^.

### Pharmacodynamics

MAC values of sevoflurane decrease with age, from 3.3% in neonates and 2.5% in infants and young adults to 1.58% to 2.05% in middle-aged adults and 1.45% in adults who are more than 70 years old ^26– 31^. In the presence of 65% nitrous oxide in the inspired gas mixture, MAC values for sevoflurane decrease by about 50% in adults ^32^.

Gender does not influence the MAC of sevoflurane, but there is some evidence suggesting that ethnic factors may play a role: MAC values reported in US studies were consistently higher (2.05% to 2.6%) ^5, 32^ than those reported for Japanese adults (1.58% to 1.71%) ^30, 31^.

### Pharmacokinetics

As for the volatile anesthetic uptake, distribution and elimination are best described by a five-compartment mammillary model ^33^. This model consists of the lungs, the vessel-rich group of organs, muscle, fat adjacent to the vessel-rich organs, and peripheral fat. In general, an inverse relationship exists between the blood/gas partition coefficient of a volatile anesthetic and the time required for the inspired and alveolar concentrations to reach equilibrium. Sevoflurane has a low blood/gas partition coefficient (0.69), resulting in a swifter equilibration of the alveolar-to-inspiratory fraction (F _A_/F _I_ ratio) than with enflurane and isoflurane but slightly slower than with nitrous oxide and desflurane ^33, 34^.

Because of its pleasant odor and the absence of irritation to the airways, sevoflurane can be used for inhalational induction both in children and in adults ^35^. Studies have shown that induction is as rapid as ^36, 37^ or even swifter than ^38– 40^ with halothane.

Elimination of a volatile anesthetic is also related to its blood solubility. Between 95% and 98% of sevoflurane is eliminated through the lung. The driving force for this elimination is the difference in partial pressures between the inspired gas mix and the pulmonary capillary blood. In humans, 2% to 5% of the absorbed dose of sevoflurane is metabolized by the liver, resulting in the formation of inorganic fluoride and the organic fluoride metabolite hexafluoroisopropanol ^41^. The latter is conjugated with glucuronic acid and excreted rapidly via the kidneys. The biotransformation of sevoflurane occurs predominantly through cytochrome P450(CYP)2E1 ^42, 43^. Serum inorganic fluoride concentrations after sevoflurane anesthesia are dose-dependent, reaching 10 to 20 µmol/L after 1 to 2 MAC hours and up to 20 to 90 µmol/L with prolonged exposure ^41^. Although most studies could not show nephrotoxic effects after sevoflurane anesthesia ^44^, some controversial reports ^45^ of mild renal dysfunction after the use of sevoflurane resulted in a recommendation by the US Food and Drug Administration for caution in the use of sevoflurane in patients with coexisting renal disease. Interestingly, the majority of data report no differences in pharmacokinetics between patients with and those without kidney diseases ^46, 47^. Percutaneous losses account for less than 1% of the total uptake of sevoflurane ^48^.

## Effects on vital systems

Like the effects of other anesthetic agents, those of sevoflurane on the vital systems are mostly depressant.

### Respiration

A decrease in ventilation leading to apnea at concentrations of between 1.5 and 2.0 MAC can be observed. The ventilatory depression with sevoflurane is the result of a combination of central depression of medullary respiratory neurons ^49^ and depression of diaphragmatic function ^50^ and contractility ^51^.

Sevoflurane provides bronchodilation and attenuates bronchial smooth muscle constriction by histamine or acetylcholine and can be safely used in patients with asthma ^21^. Hypoxic pulmonary vasoconstriction is inhibited by sevoflurane in a dose-dependent manner and is not mediated by cyclo-oxygenase ^21– 23^.

### Circulation

Sevoflurane decreases blood pressure in a dose-dependent manner by decreasing total peripheral resistance. At clinically relevant concentrations, cardiac output is usually preserved ^21– 23^. Heart rate remains unchanged or even decreases. Coronary blood flow remains preserved and regional blood flow to other vascular beds appears to be maintained at least when systemic hemodynamics are preserved. For sevoflurane (unlike for desflurane), no sympathetic nervous system activation is observed ^21– 23^. Although sevoflurane has been reported to prolong the QT and the QTc interval ^52^, it has no effect on the normal cardiac conduction pathways and therefore is considered a safe agent that can also be used in cardiac electrophysiological procedures ^25^.

### Central nervous system

Sevoflurane is a cerebral vasodilator. In neurosurgical patients, sevoflurane decreased middle cerebral artery flow velocity and caused no epileptiform electroencephalogram activity and no increase of intracranial pressure ^53^. Cerebral autoregulation is maintained at low concentrations of sevoflurane ^54^, but higher doses seem to decrease autoregulatory capacity ^55^.

## Safety

Overall, sevoflurane is considered to be a safe and reliable agent that has also been used in uncommon medical conditions such as pheochromocytoma, acute intermittent porphyria, carnitine deficiency, muscular dystrophy, multiple sclerosis, primary aldosteronism, and myotonic dystrophy ^23^.

In the early years of its use, a number of reports on malignant hyperthermia with sevoflurane were published. In many of them, it was difficult to isolate the potential effects of sevoflurane from the influence of the concurrent use of other triggers such as succinylcholine. Animal studies have suggested that the malignant hyperthermia trigger of sevoflurane was substantially lower than that of other volatile anesthetic agents ^56^. However, a recent Japanese database study did not find evidence that sevoflurane would be a weaker triggering agent for malignant hyperthermia ^57^. Since its introduction in clinical practice, sevoflurane has been safely used in millions of people, and reports of sevoflurane-related malignant hyperthermia are scarce. Nevertheless, it seems wise to avoid exposure to sevoflurane in patients with a known susceptibility.

In the early years of clinical sevoflurane use, it was reported that sevoflurane in the presence of the CO _2_ absorbers soda lime (calcium, sodium, and potassium hydroxide mixture), or baralyme (barium, sodium, calcium, and potassium hydroxide mixture) degrades to compound A. This degradation occurs in the anesthesia machine as a result of the extraction of an acidic proton (from the inhalational anesthetic) by a strong base (soda lime or baralyme) ^58^. The rate of degradation at a given temperature and moisture level is two to four times greater with baralyme compared with soda lime ^58, 59^. The order of solubility of inhalation anesthetics in dry soda lime is sevoflurane > enflurane > desflurane ≥ halothane > isoflurane ^4^. As a consequence, more sevoflurane is absorbed into the CO _2_ absorber than is observed with other inhalational anesthetics. The production and subsequent inhalation of compound A correlate inversely with the inflow rate ^60^ and directly with the absorbent temperature ^61^. In addition, low fresh gas flows of sevoflurane are associated with increased temperatures in the CO _2_ absorber ^62^. Therefore, compound A production can be limited by decreasing the temperature of the absorbent ^63^. For these reasons, US and Canadian package labels and licensing authorities have recommended minimal fresh gas inflow rates of 1 or 2 L/min, although other licensing authorities have not made such a recommendation. Compound A production can also be reduced by the amount of absorbent (smaller canisters) ^64^ and adapting the composition of the absorbent by eliminating potassium and sodium hydroxide ^65– 67^. The clinical implications of compound A production have been a point of debate for many years ^68^, but the introduction of the new-generation absorbers ^11^ has made this issue largely obsolete.

## Special populations

### The pediatric patient

There seems to be no significant difference in sevoflurane pharmacokinetics between children and adults ^24^. Because of its pleasant odor, lack of airway irritation, and maintenance of stable hemodynamics, sevoflurane is the agent of choice for mask induction. In general, complications upon emergence are infrequent, although some studies mention a significantly higher incidence of excitement/agitation with sevoflurane ^69, 70^. However, this observation has been linked to the fact that the prompt recovery from anesthesia with sevoflurane also facilitates earlier awareness of postoperative pain ^69, 71^. This causal relationship was confirmed in a number of studies demonstrating that adequate pain treatment was associated with significantly fewer episodes of emergence agitation ^72, 73^.

### The ambulatory patient

Ambulatory surgery has increased rapidly in recent years and this has put an emphasis on the use of short-acting drugs in anesthetic practice, allowing fast recovery and early mobilization. Differences in early recovery between sevoflurane, desflurane, and propofol have been reported to be small but in favor of the inhaled anesthetics ^74^, although the clinical implications of these small differences are debatable. Postoperative nausea and vomiting are higher with volatile anesthetics than with propofol, but adequate anti-emetic prophylaxis can prevent or blunt this side effect.

### The obese patient

The prevalence of obesity is increasing dramatically, not only in industrialized countries but also in developing ones. As a consequence, we encounter a growing number of morbidly obese patients who need different types of surgery. Obese patients are traditionally reported to have slower emergence from anesthesia because of a delayed release of volatile anesthetics from the excess fat tissue. However, comparable recovery times have been reported in obese and non-obese subjects after anesthetic procedures lasting 2 to 4 hours ^75^.

The new inhalation drugs have a much lower lipid solubility compared with the older volatile anesthetic agents, resulting in a more rapid and consistent recovery profile ^76^. For sevoflurane, no significant differences in F _A_/F _I_ ratio have been observed, but the wash-out curve—that is, the alveolar-to-expiratory fraction (F _A_/F _AO_ ratio)—was reported to be slower in obese patients compared with non-obese patients. However, 5 minutes after sevoflurane discontinuation, no differences in wash-out were observed between obese and non-obese patients ^77^. Several studies have compared kinetic profiles of sevoflurane and desflurane. Some studies observed more rapid emergence from anesthesia with desflurane but this was not confirmed in other studies (reviewed in 78). Finally, an advantage of sevoflurane in this setting is that it allows progressive induction of anesthesia via face mask ^79^.

## Organ protection

### The heart

The finding in the late 1990s that sevoflurane was capable of limiting the extent of myocardial infarction after myocardial ischemia triggered a new research direction investigating potential cardioprotective effects of volatile anesthetic agents. Experimental studies have clearly indicated that volatile anesthetic agents are capable of protecting the myocardium against the consequences of ischemia-reperfusion injury by decreasing the extent of myocardial damage, decreasing the extent of reperfusion injury, and better preserving myocardial function. Subsequent research was directed toward unraveling the underlying mechanisms and intracellular pathways of these cardioprotective effects ^80– 85^.

Although the experimental evidence of cardioprotection with volatile anesthetic agents was quite straightforward, the implications for clinical practice remain a point of debate. The potential cardioprotective effects related to the use of volatile anesthetics were first explored in the setting of cardiac surgery. In coronary artery surgery patients, the results of preconditioning protocols were conflicting: some authors demonstrated a protective effect whereas others failed to observe such an effect. Later, it became clear that this might be attributed to the preconditioning protocol used. It also seems that the administration of the volatile anesthetic agent throughout the entire procedure results in a more pronounced protective effect than when administered intermittently. It is beyond the scope of this review to discuss these studies in detail. The interested reader is referred to a number of reviews on the topic ^86– 95^.

For non-cardiac surgery, the potential clinical implication of the cardioprotective properties of volatile anesthetics is even more debatable. One small study in vascular surgery patients observed a lower incidence of cardiac complications in patients treated with sevoflurane compared with those anesthetized with propofol ^96^. Others, however, observed no difference in the extent of myocardial damage when comparing a volatile anesthetic regimen with a total intravenous regimen ^97, 98^. This clearly indicates that only in the presence of myocardial ischemia/reperfusion injury can a potential beneficial effect of volatile anesthetics be expected ^99^. Interestingly, in the study by Lurati Buse and colleagues ^98^, in which 385 patients were randomly assigned to receive anesthesia with either sevoflurane or propofol, the incidences of perioperative myocardial ischemia were comparable (40.8% in the sevoflurane group and 40.3% in the propofol group). Within 12 months, 14 patients had a major cardiac event in the sevoflurane-treated group (7.6%) and 17 in the propofol-treated group (8.5%). However, given that a potential cardioprotective effect of volatile anesthetic agents relates to a modulation of the extent of myocardial ischemia/reperfusion injury, the analysis of the major cardiac events needs to be focused on the occurrence of these events in the subgroup of patients who had perioperative myocardial ischemia. To further clarify this issue, the authors performed an additional analysis of their data. They found that the incidence of major cardiac complications in patients with evidence of perioperative myocardial ischemia was similar in both groups: 8 out of 67 patients (11.9%) in the sevoflurane group and 9 out of 72 patients (12.9%) in the propofol group. The remaining 6 and 8 patients with postoperative major cardiac complications had not shown any evidence of perioperative myocardial ischemia (Seeberger M, unpublished observations).

### Other organ systems

Clinical studies on the protective effects of sevoflurane on other organ systems are scarce and limited to a small number of patients. Three studies from the same group suggest protective effects after liver ^100, 101^ and lung ^102^ ischemia with sevoflurane, but the potential implications on long-term outcome remain to be established.

## Neurotoxicity in the young and aged brain

Preclinical evidence in rodents and non-human primates has caused concern regarding the safety of anesthesia in infants and children. Indeed, animal studies suggest that neurodegeneration with possible cognitive sequelae may constitute a potential long-term risk of anesthesia in neonatal and young pediatric patients (reviewed in 103, 104). No hard clinical data suggest that the use of anesthetics in the neonate or young child is associated with signs of developmental neurotoxicity ^103^. It has been argued that the increased risk of poor outcome in some human cohort studies is because of the inflammation and stress associated with the surgery rather than the anesthetic ^105^. It is expected that the results of two ongoing large-scale studies—the Multi-site Randomized Controlled Trial comparing Regional and General Anesthesia for Effects on Neurodevelopment Outcome and Apnea in Infants (GAS) study and the Pediatric Anesthesia and NeuroDevelopment Assessment (PANDA) study—will give more insight into the problem ^104^.

Similarly, experimental studies, observing effects of anesthetic agents on memory formation and the induction of neurodegenerative changes on a cellular level, have raised concerns about the effects of anesthesia and surgery on the elderly brain. The incidence of postoperative cognitive dysfunction (POCD) varies according to the definitions used in the various studies but is reported to be higher in major surgery (reviewed in 106– 108). Whether general anesthesia contributes to POCD remains uncertain. A recent meta-analysis of 26 randomized trials comparing general to regional anesthesia was unable to identify general anesthesia as an independent risk factor for POCD ^109^. It is conceivable that surgical trauma and underlying pathology are of greater importance. Given the complexity and still-unknown elements of the pathogenesis of POCD, further research on the topic is needed.

## Conclusions

Since its introduction in clinical practice, sevoflurane has gained wide acceptance as an anesthetic for various types of surgery. Its ease of administration, versatility, and stable hemodynamic profile make it a safe and easily applicable anesthetic agent.
