# Supplementary material for: Sevoflurane
Source: F1000Res. 2015 Aug 25;4(F1000 Faculty Rev):626. [Version 1] doi: 10.12688/f1000research.6288.1 (PMC4560253; doi:10.12688/f1000research.6288.1)
Supplement: Peer review history

# Peer review: Referee response for version 1

**DOI:** 10.5256/f1000research.6744.r10111

**Published:** 2015-08-25

**Authors:** Manfred Seeberger

**Affiliations:** 1 Department of Anaesthesiology and Intensive Care Medicine, Klinik Hirslanden, Witellikerstrasse 40, Zurich, 8032, Switzerland

I have read this submission. I believe that I have an appropriate level of expertise to confirm that it is of an acceptable scientific standard.

# Peer review: Referee response for version 1

**DOI:** 10.5256/f1000research.6744.r10110

**Published:** 2015-08-25

**Authors:** Roderic Eckenhoff

**Affiliations:** 1 Department of Anesthesiology and Critical Care, University of Pennsylvania, Philadelphia, PA, 19104-6112, USA

I have read this submission. I believe that I have an appropriate level of expertise to confirm that it is of an acceptable scientific standard.
